# Neuronal Activity Regulating the Dauer Entry Decision in *Caenorhabditis elegans*

**DOI:** 10.1523/ENEURO.0447-24.2025

**Published:** 2026-01-15

**Authors:** Sharan J. Prakash, Maedeh Seyedolmohadesin, Mark G. Zhang, Sarah M. Cohen, Shahla Gharib, Vivek Venkatachalam, Paul W. Sternberg

**Affiliations:** ^1^Division of Biology and Biological Engineering, California Institute of Technology, Pasadena, California 91125; ^2^Physics Department, Northeastern University, Boston, Massachusetts 02115

**Keywords:** *Caenorhabditis elegans*, dauer, decision-making, memory, neurobiology, neuroscience

## Abstract

The life cycle of the model nematode *Caenorhabditis elegans* involves a choice between two alternative developmental trajectories. Hermaphroditic larvae can either become reproductive adults or, under conditions of crowding or low food availability, enter a long-term, stress-resistant diapause known as the dauer stage. Chemical signals from a secreted larval pheromone promote the dauer trajectory in a concentration-dependent manner, and their influence can be antagonized by increased availability of a microbial food source. The decision is known to be under neuronal control, involving both sensory and interneurons. However, little is known about the dynamics of the underlying circuit, and the circuit mechanisms by which short-term fluctuations in the ratio of food and pheromone experienced by individual larvae are remembered and averaged over several hours. To investigate this, we quantitatively characterized the neuronal responses to food and pheromone inputs by measuring calcium traces from ASI and AIA neurons, each of which has previously been implicated in regulation of dauer entry. We found that calcium in ASI increases linearly in response to food and similarly decreases in response to pheromone. Notably, the ASI response persists well beyond removal of the food stimulus, thus encoding a memory of recent food exposure. In contrast, AIA reports instantaneous food availability and is unaffected by pheromone. We discuss how these findings may inform our understanding of this long-term decision-making process.

## Significance Statement

The neural circuit underlying the decision to enter the dauer diapause in *Caenorhabditis elegans* is poorly understood. In particular, it is unclear how fluctuating inputs with opposite effect on the decision are averaged and remembered over several hours to allow accurate decision-making. In this study, we show that a single sensory neuron responds oppositely to chemical cues with opposite effects on the decision and retains a memory of recent input in a persistent calcium transient. These findings demonstrate how microfluidics and live imaging, combined with neuronal silencing and behavior assays, can be leveraged to understand the dynamics of the neural circuit regulating the decision.

## Introduction

**Figure 1.**
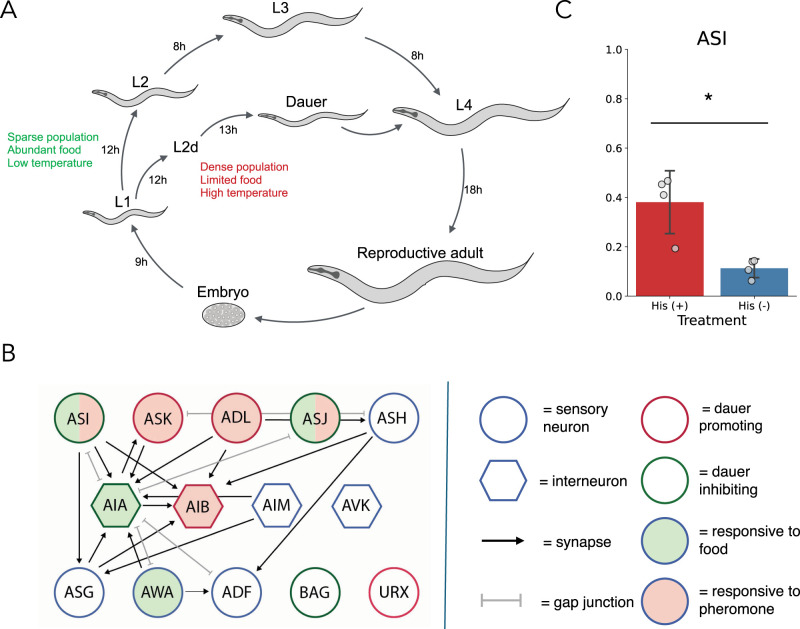
Neuronal control of *C. elegans* dauer entry. ***A***, *C. elegans* life cycle. Larvae can enter the dauer stage after L1 under unfavorable conditions and return to L4 when conditions improve. The progression to dauer involves an uncommitted L2d phase where larvae can escape dauer entry to L3, while committed dauers can only progress to L4, i.e., dauer is an alternative L3. ***B***, Neural circuit diagram of all neurons mentioned in this paper. Sensory neurons are circles, and interneurons are hexagons. Outline colors refer to the valence of a neuron's activity on dauer entry (green, promotes adult development; red, promotes dauer entry; blue, no reported effect). Specifically, ASK and ADL promote dauer entry (Chai et al., 2022b), as do AIB (Chai et al., 2021), URX (Chai et al., 2022a), and ASJ (Schackwitz et al., 1996). Dauer-inhibiting neurons are ASI (Bargmann and Horvitz, 1991; this study) and BAG (unpublished). Fill colors refer to response of the neuron to chemical inputs as evidenced by calcium imaging (green, responsive to food; red, responsive to pheromone; clear, no reported effect). Neurons exhibiting a calcium response to food are AWA (Larsch et al., 2015), AIA (this study), ASI (Chalasani et al., 2007; this study), and ASJ (Zhang et al., 2024). In the case of ASI and ASJ, the combined green and red fill indicates that food responses are inhibited by pheromone [this study and Zhang et al. (2024), respectively]. Arrows refer to developmentally stable chemical synapses, while gray double-headed edges refer to gap junctions. Only connections including at least three chemical synapses and two gap junctions are included, for life stages embryo through L2. All circuit connectivity adapted from nemanode.org (Witvliet et al., 2021). ***C***, Inhibition of ASI promotes dauer entry. Animals expressing HisCl1 in individual neurons were grown on plates with [His (+)] and without histamine [His (−)]. *N* = 4 population assays (80–100 worms each) per treatment. Stars (*) denote Student's *t* test (*p* < 0.05). Data represented as mean with SEM.

Phenotypic plasticity allows many organisms to undergo adaptive developmental and physiological changes in response to changing environmental conditions. The most dramatic forms of phenotypic plasticity are polyphenisms, in which individuals are able to choose between discrete alternative morphologies (Nijhout, 2003). A well-known polyphenism in nematodes is the dauer diapause, an alternative developmental trajectory available to *Caenorhabditis elegans* larvae after the first (L1) larval stage (Fig. 1*A*; Cassada and Russell, 1975). Larvae enter dauer under adverse environmental conditions and survive for several months without food (Klass and Hirsh, 1976). When conditions improve, larvae can resume normal development at the L4 stage, without any effect on adult lifespan. The primary chemical inputs to the decision are microbial food signals, which promote adult development, and the dauer pheromone—a cocktail of secreted small molecules that promote dauer entry by providing a proportional signal of local crowding (Golden and Riddle, 1982, 1984b).

Through laser ablation and genetics experiments, entry into the dauer diapause was shown to be under nervous system control (Albert et al., 1981; Bargmann and Horvitz, 1991; Schackwitz et al., 1996; Zwaal et al., 1997). Despite this early advance, our understanding of the neuronal activity dynamics regulating the decision lags well behind our understanding of the molecular pathways involved (Fielenbach and Antebi, 2008). The sophistication of neural circuit control is likely to be crucial in dauer regulation, due to the complexity of the task at hand. Previous work has shown that, rather than briefly assessing conditions immediately prior to the L2 molt, larvae are influenced by pheromone exposure for several preceding hours during L1. When larvae were shifted from liquid media lacking pheromone into high pheromone media after various durations, earlier shifts resulted in increased dauer entry (Schaedel et al., 2012). Similarly, larvae cultured with low levels of pheromone insufficient for dauer entry exhibit a pronounced delay in the L2 molt, consistent with a prolonged period of evidence accumulation, known as L2d (Fig. 1*A*; Golden and Riddle, 1984a).

In nature, the distribution of food and pheromone is nonuniform, and the food-to-pheromone ratio experienced by larvae as they navigate local environments is likely to fluctuate on a timescale of seconds to minutes. To correctly anticipate future conditions and influence development accordingly, the nervous system must collect and compare fluctuating patterns of chemical inputs during these early larval stages, emphasizing long-term trends. This suggests the existence of a distributed neural circuit capable of considerable plasticity and memory and implies one or more mechanisms that can bridge short timescale of environmental inputs to the hours-long timescale of the decision. Understanding these aspects of the decision requires detailed study of the activity dynamics of the neurons involved.

Technological developments have provided new opportunities to understand the neural circuit governing dauer entry in detail. Recently, chemical silencing of individual neurons (Pokala et al., 2014) in combination with population assays were used to screen for neurons involved in dauer entry, demonstrating a method to systematically implicate individual neurons in the behavior and elucidate circuit composition (Chai et al., 2022b). In addition, microfluidics and calcium imaging experiments revealed that ASK and ADL sensory neurons are activated by pheromone (Chai et al., 2022b). Thus, a combined approach employing neuroanatomy, genetics, chemical silencing, behavior assays, and calcium imaging can provide a comprehensive and increasingly quantitative picture of the neuronal regulation of dauer entry, from environmental inputs, to neural circuit dynamics, to behavior.

Among the neurons previously implicated in dauer regulation by genetic and laser ablation approaches are the sensory neurons ASI and ASJ (Fig. 1*B*). ASI expresses several pheromone receptors (McGrath et al., 2011; Park et al., 2012) and exhibits a calcium response to soluble chemicals present in liquid OP50 solution (Calhoun et al., 2015). Both ASI and ASJ synthesize peptides that inhibit dauer formation in the presence of food, but not in the presence of pheromone (Ren et al., 1996; Murakami et al., 2001; Li et al., 2003; Cornils et al., 2011). Eliminating the ion channel *tax-2* also inhibits expression of some insulins in ASI, suggesting that their induction is activity dependent (Artan et al., 2016). ASJ is known to be involved in dauer exit, but its role in dauer entry is less clear. For instance, laser ablation experiments suggest that ASJ has dauer-promoting effect during the early larval stages (Schackwitz et al., 1996), potentially via expression of dauer-promoting neuropeptide *ins-1* (Pierce et al., 2001). More recent imaging studies have revealed complex response dynamics of the ASJ neuron to food input, as well as inhibition of the food-induced calcium response by pheromone (Zhang et al., 2024). Another neuron whose connectivity is suggestive of a role in food-pheromone integration is the interneuron AIA. AIA is postsynaptic to the food sensor AWA (Larsch et al., 2015), as well as pheromone-sensing ADL and ASK neurons, suggesting a neural pathway for its previously observed inhibition by pheromone (Chai et al., 2022b). Chemical silencing experiments previously showed that AIA inhibition promotes dauer entry (Chai et al., 2022b).

Overall, these studies point to ASI and ASJ as key sites for the integration of sensory cues with opposite effects on dauer and potentially a role for AIA. However, no study has previously investigated the calcium activity of ASI or AIA in response to combinations of these cues. We found that the calcium activity of ASI displays a striking memory of food exposure and is oppositely influenced by food and pheromone, while AIA reports the immediate availability of food and is not responsive to pheromone. This suggests specialization of the sensory neuron ASI as a cellular site of antagonism between the key chemical inputs in the dauer decision and provides evidence that the memory mechanism required to bridge the timescales of sensory perception and commitment involves a long-lived calcium signal. Our results also contrast with previous studies which describe the comparison of sensory inputs of opposite valence at the interneuron level (Ghosh et al., 2017; Gat et al., 2023).

## Materials and Methods

### Animal maintenance and strains

Animals were cultivated at 21°C on standard nematode growth media (NGM) plates seeded with *E. coli* OP50 cultured in lysogeny broth (LB). All cGAL strains were generated by crossing UAS effector and GAL-4 driver strains previously described (Wang et al., 2017; Nava et al., 2023). The strain for imaging AIA (Chai et al., 2022b) and the strain used to image ASI (Lin et al., 2023) were previously published. The full list of strains used in this study is available in Table 1.

**Table 1.** List of strains and associated genotypes used in this study

Strain number	Description	Transgenes	Genotype	Origin	Source	Publication	Availability
PS7199	Histamine Chloride Ion Channel	syIs371	*syIs371*[*UAS::HisCL::SL2::GFP*, P*unc-122::GFP*]	Existing strain	Sternberg lab	Wang et al. (2017)	CGC
PS9034	ASI driver	syIs686	*syIs686[Pgpa-4-GAL4(sk)-VP64, Punc-122p::RFP]*	Existing strain	Sternberg lab	Nava et al. (2023)	CGC
PS9190	ASI::His-Cl	syIs371; syIs686	*syIs371[UAS::HisCL::SL2::GFP, Punc-122::GFP]; syIs686 [Pgpa-4-GAL4(sk)-VP64 Punc-122::RFP]*	Crossed PS7199 x PS9034	Sternberg lab	This paper	Upon request
PS9111	AIA GCaMP strain	syIs761	*syIs761[Pgcy-28.d::GCaMP6s, Pmyo-2::mCherry]*	Existing strain	Sternberg lab	Chai et al. (2022a)	Upon request
ZM10104	pan-sensory neuronal GCaMP	aeaIs008; hpIs728	*aeaIs008[Pift-20:: GCaMP6s::3xNLS]; hpIs728[Pgpc-1::wCherry]*	Existing strain	Sternberg lab	Lin et al. (2023)	Upon request
PS8720	His-Cl inhibitor expressed in muscle neuron	Kp1368; syIs371	*Kp1368 [myo-2P::NLS::mCherry]; syIs371[15xUAS::HisCL::SL2::GFP::let-858 3'UTR]*	Existing strain	Sternberg lab	Wan et al. (2025) (bioRxiv)	Upon request

### Pheromone preparation

Crude pheromone was prepared as described previously (Schroeder and Flatt, 2014). Briefly, *C. elegans* were cultured in flasks with OP50 *E. coli* over several days until starved, with one additional round of feeding and starvation. Worms were separated from liquid by centrifugation and filtration. Crude pheromone was isolated by heating and ethanol extraction and resuspended in water.

### Dauer assays

Dauer assays were performed as described previously (Lee et al., 2017; Chai et al., 2022b). On Day 1, histamine chloride inhibition plates were prepared as follows. For each plate, 2 ml of peptone-free Nematode Growth Medium (NGM) was mixed with either 6 or 10 μl of crude pheromone extract. For experimental plates, 30–40 mg of histamine dihydrochloride powder was first dissolved in 15 ml of NGM solution at 60°C, while control plates lacked histamine. Four plates were prepared per treatment for each experiment. One control plate lacking pheromone, but containing histamine, was also prepared for each experiment from the same histamine (+) NGM dilution. Roughly 100 L4 adult hermaphrodites were picked onto new seeded plates, and a single colony of OP50 was used to inoculate LB cultured overnight at 37°C. On Day 2, OP50 was concentrated to 8% w/v in S-basal. Then, 2 μl of this OP50 was added to each plate to allow 80 adults to lay 70–90 eggs at 25°C. As a control, the line PS8720, expressing the His-Cl effector transgene under the *myo-3* promoter was transferred to the histamine (+) control plate. Successful inhibition results in cessation of locomotion in these animals. Remaining OP50 was heat killed at 95–100°C for 10 min and 18 μl added per plate. Once dried, all plates were parafilmed and moved to 25°C for 72 h. Dauer and non-dauer worms were counted for each plate.

### Statistical analysis

The Student's *t* test was used to test for statistically significant difference in the dauer fraction between histamine added and histamine absent treatments, using the numpy python package. Original data for the dauer assay in Figure 1 are listed in Table 2.

**Table 2.** Dauer assay involving chemical inhibition of ASI neuron via histamine-chloride silencing

Neuron	Plate	Treatment	Dauers	Non-dauers	Dauer fraction
ASI	1	His (+)	29	35	0.453
ASI	2	His (+)	28	32	0.467
ASI	3	His (+)	11	46	0.193
ASI	4	His (+)	32	46	0.41
ASI	5	His (−)	7	59	0.106
ASI	6	His (−)	8	49	0.14
ASI	7	His (−)	5	76	0.062
ASI	8	His (−)	11	66	0.143

### Microfluidic device fabrication

We designed a two-layer microfluidic chip capable of delivering sequences of stimuli with a worm trap suitable for housing worms at L4 larval stage. The chip was designed in AutoCAD software and sent to Artnet Pro for photomask printing. Photolithography in a clean room was performed on a silicon wafer to make the two-layer mold from the photomask. For the first layer, which included the worm trap, SU-8 2025 was spin coated on the silicon wafer at 4,000 rpm to achieve 25 μm thickness. For the second layer, the same photoresist was spin coated at 1,250 rpm for a thickness of 70 μm. Polydimethylsiloxane (PDMS) was poured over the mold and cured on a 90°C hotplate to solidify. Each PDMS chip was then punched with a 1 mm biopsy punch and was bonded to a coverslip using a handheld corona treater.

Calcium imaging L4 stage animals were assayed and placed in the microfluidic device. For imaging of AIA, a strain expressing GCamp6s specifically in AIA was used (PS9111). For imaging of ASI, a strain expressing GCamp6s in all sensory neurons was used (ZM10104). For each experiment, *E. coli* OP50 was cultured overnight in LB, and its supernatant was collected. Crude pheromone extract was diluted to a concentration of 2.5% (v/v) in either buffer (H_2_O) or OP50 supernatant. Each stimulus was delivered to the animal's nose for a duration of 15 s, followed by either buffer (H_2_O) or pheromone. Fluorescence was recorded with a spinning disc confocal microscope (Dragonfly 200, Andor) and a sCMOS camera (Photometrics Kinetix). The fluorescence was captured from GCaMP6s at a rate of 10 ms per 1.0 μm z-slice, with 25 z-slices per volume and 4 volumes per second. To extract calcium activity from the recorded data, we performed the following steps: (1) Background intensity was subtracted from each recorded volume. (2) The center of the ROI was annotated at one timepoint, and then the center was tracked throughout the entire recording using the ZephIR tracking algorithm (Ryu et al., 2024). For ASI neurons, the ROI was defined as the neuronal nucleus, and for AIA neurons, the ROI was defined as the processes located in the gap junction. 4. Average pixel intensity from each ROI was calculated. Δ*F*/*F*0 was computed, where *F*0 was defined as the average intensity during the 5 s window preceding stimulus delivery.

## Results

We first sought to confirm the direction of ASI influence on dauer entry via chemical silencing and found that silencing ASI promotes dauer entry as expected (Fig. 1*C*). To examine the quantitative features of the associated neuronal responses, we next performed calcium imaging experiments on ASI and AIA. Microfluidic devices that can house L1 larvae are still under development; however L4 larvae are well suited to existing setups. We delivered food and pheromone to L4 larvae immobilized in a microfluidic chip and imaged the calcium dynamics of each of these neurons using GCaMP6s.

**Figure 2.**
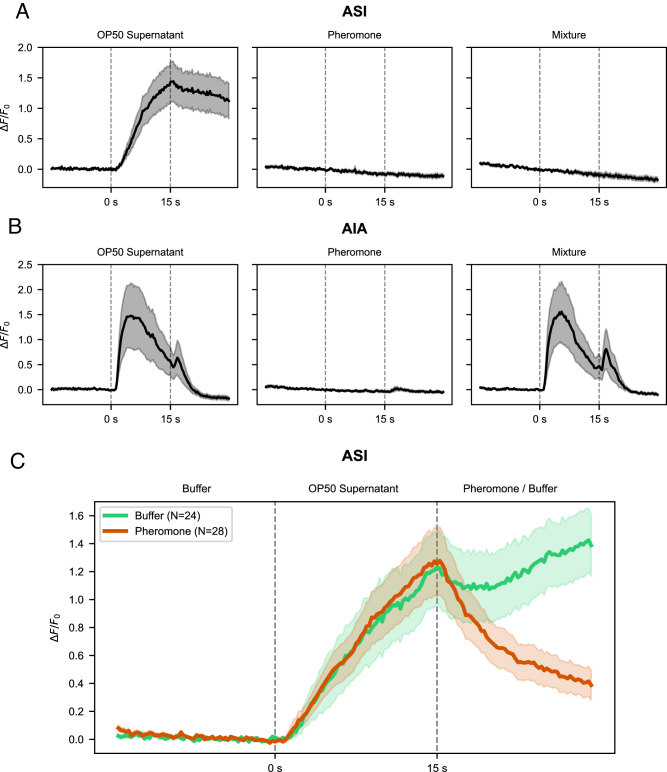
Calcium responses of ASI and AIA neurons to liquid inputs. L4 larvae were loaded into a microfluidic chip allowing exposure of sensory neurons each stimulus for 15 s. ***A***, ASI exhibits slow dynamics in response to food (OP50 supernatant), with the calcium signal persisting beyond food removal (left panel), but no calcium response is seen for crude pheromone (middle panel). Mixture of food and pheromone eliminates the food response (right panel). ***B***, AIA calcium signal responds immediately to food (left panel), but not pheromone (middle panel). Mixture of crude pheromone and food results in a normal food response (right panel). ***C***, ASI calcium response to bacterial food and pheromone applied in series. L4 larvae were exposed as before to OP50 supernatant for 15 s (both green and red lines), followed by exposure to crude pheromone extract for 15 s (red line only). Exposure to OP50 results in a progressive increase in calcium signal, which is reversed by addition of pheromone. In the control group, 15 s of OP50 exposure is followed by 15 s of buffer (green line). The activated state persists for at least 15 s after the removal of OP50.

We found that ASI responds strongly to OP50 supernatant, such that the amount of intracellular calcium increases linearly with stimulus duration (Fig. 2*A*, left panel). Notably, the calcium signal in ASI persisted for at least 15 s after the stimulus was removed. Crude pheromone in isolation elicited no response in either neuron (Fig. 2*A*, middle panel; Fig. 2*B*, middle panel). We found that adding crude pheromone to the food stimulus eliminated the food response in ASI (Fig. 2*A*, right panel). Finally, by applying successive food and pheromone stimuli, we found that the calcium signal in ASI induced by food could be antagonized by subsequent exposure to pheromone (Fig. 2*C*), showing that although stimulation by food can induce long-lasting effects on its calcium state, ASI remains highly sensitive to instantaneous presentation of pheromone immediately following food exposure. In contrast to ASI, AIA responded quickly to OP50 exposure (Fig. 2*B*, left panel) and showed no response to pheromone in isolation (Fig. 2*B*, middle panel). Previous work had suggested that pheromone inhibits spontaneous AIA activity (Chai et al., 2022b), and other studies have shown that this neuron is capable of sensory integration in other contexts (Dobosiewicz et al., 2019). Therefore, we examined the possible inhibition of AIA by pheromone by mixing food and pheromone inputs. We saw no suppression of the AIA food response by pheromone (Fig. 2*B*, right panel), suggesting that AIA may not be highly sensitive to pheromone input from sensory neurons.

## Discussion

The role of the nervous system in regulating dauer entry was recognized in the early 1980s (Albert et al., 1981). Over the following decades, a variety of experimental methods—including laser ablation (Bargmann and Horvitz, 1991; Schackwitz et al., 1996), molecular genetics and biochemistry (Kenyon et al., 1993; Ren et al., 1996; Murakami et al., 2001; Kim et al., 2009; Park et al., 2010, 2012; Cornils et al., 2011), and analytical chemistry (Pungaliya et al., 2009)—were used to identify the key pathways and small molecules involved [for comprehensive reviews, see Butcher (2017) and Fielenbach and Antebi (2008)]. More recently, the development of microfluidics, live imaging, and chemical silencing techniques have enabled exploration of the neural circuit dynamics regulating dauer (Chai et al., 2022b; Zhang et al., 2024). Here, we extend this approach to investigate two neurons implicated in the response to food and pheromone, the two primary chemical inputs in the decision.

In addition to demonstrating the opposite effects of food and pheromone on the calcium activity of ASI, we report the persistence of an “ON” state in this neuron following removal of food stimulus. We suggest that this is a memory mechanism that allows the short timescale of fluctuations in sensory input and the long timescale of the decision to be bridged. This provides a more satisfying explanation than other possible models. In principle, memory could reside in long-lived neuropeptides whose production is induced by food perception or in gene regulatory networks that allow their persistent synthesis. However, in the absence of continuous neuronal activation, these peptides cannot be continuously secreted from ASI. Intermittent neuropeptide release would likely result in inefficient signaling due to diffusion away from receptors in downstream tissues. We note that other mechanisms, such as changes in synaptic weight, may also be important in bridging the timescales of neuronal activity and the physiological decision. Secondly, given that the food-induced calcium signal in ASI can be immediately antagonized by pheromone, the ASI response profile suggests an averaging mechanism by “integration over time,” i.e., the calcium response profile is proportional to the integral of historical differences between food and pheromone exposure. Though we anticipate that the full circuit will involve a number of connected neurons and sensory modalities, this result highlights the capacity for individual neurons in the compact *C. elegans* nervous system to perform nontrivial computations. It will be of interest to see explore how differences in the duration, magnitude, and frequency of incoming food-derived cues influence the persistence and magnitude of the ASI response and how prior conditioning by pheromone may affect its sensitivity to food.

The rapid adaptation of AIA to OP50 extract is consistent with its documented response to the odor diacetyl (Dobosiewicz et al., 2019) and its known role in chemotaxis (Larsch et al., 2015). We speculate the primary role of AIA in dauer entry may be to transduce volatile signals produced by bacteria, sensed by presynaptic neurons such as AWA (Larsch et al., 2015). This also raises the question of how pheromone-sensing neurons ADL and ASK promote dauer entry. It will be informative to examine the effect of their activity on ASI and ASJ, for instance, through combined optogenetics and calcium imaging experiments.

Going forward, studies of the dauer circuit will benefit from the use of calcium imaging from neuronal subsets to describe the flow of sensory information through the nervous system. Along with long-term neuronal silencing experiments, these data can be tied to behavior. These data will help uncover the mechanisms and algorithms that regulate the decision to enter dauer.

## Synthesis

Reviewing Editor: David Schoppik, New York University Grossman School of Medicine Decisions are customarily a result of the Reviewing Editor and the peer reviewers coming together and discussing their recommendations until a consensus is reached. When revisions are invited, a fact-based synthesis statement explaining their decision and outlining what is needed to prepare a revision will be listed below. The following reviewer(s) agreed to reveal their identity: Niels Ringstad.

1. As written, we think the manuscript would be stronger for a clear acknowledgement that many conclusions of this study recapitulate conclusions of other studies. Specifically, we recommend that the revision more candidly discuss prior work from the Sternberg lab that highlights contributions from AIA interneurons and non-ASI sensory neurons, including BAGs.

2.The manuscript repeatedly refer to calcium signals as 'depolarization.' This should be corrected.

3. Data showing effects of neuronal silencing on dauer-entry indicate that the baseline frequency of dauer-entry fluctuates enormously. Histamine-minus controls should show comparable rates of dauer entry, but they do not. If there is a reason for these fluctuations, it should be clearly explained. If not, the experimental approach should be revised. Our contention was that in the absence of the trigger for their chemogenetic system (histamine), the response should be the same regardless of where the chemogenetic actuator (HisCl) is expressed. That's not the case.

4. The manuscript does not sufficiently address how it contributes to the study of the dauer decision. At first glance, this study seems to largely confirm results published previously. The authors should make clear what is new.

5. We are a little confused by the statistical analysis in Table 1 vs. Figure 2. It does not appear the asterisks in Figure 2 have been corrected for multiple comparisons as in Table 1. Shouldn't only BAG be significant? If so, the manuscript should make this caveat clearly in the text.

Minor comments:

Figure 1B: Adding a color and polygon legend would make it easier for the reader to understand the figure.

Figure 3: The identifiers A-C are missing in the figure.

Table 1: Benjamini-Hochburg should be "Benjamini-Hochberg"

Line 94: The authors need to upload these notebooks to a suitable repository.

Line 110: Cultured overnight at 37C.

Line 119: We presume you mean the ROI was the nucleus, not nucleolus?

Line 164: The manuscript should clarify that imaging was on L4 stage worms. We realize imaging L2 worms is notoriously difficult, but it might be worth explaining to a non-nematode researcher the choice to image L4 worms that are past the dauer stage.

In the discussion on AIA, the Dobosiewicz et al paper (https://doi.org/10.7554/eLife.50566 ) should be mentioned, since AIA has a known role as a sensory integration neuron, which is likely more responsive to complex odor mixtures such as food.
